# AUDISTIM^®^ Day/Night Alleviates Tinnitus-Related Handicap in Patients with Chronic Tinnitus: A Double-Blind Randomized Placebo-Controlled Trial

**DOI:** 10.3390/audiolres14020031

**Published:** 2024-04-10

**Authors:** Didier Portmann, Marie José Esteve-Fraysse, Bruno Frachet, Florent Herpin, Florian Rigaudier, Christine Juhel

**Affiliations:** 1 Institut G PORTMANN, 114, Avenue d’Arès, 33000 Bordeaux, France; d.portmann@wanadoo.fr; 2 Centre d’Exploration Fonctionnelle d’Otoneurologie, 10, rue Falguière, 75015 Paris, France; frayssemariejose@gmail.com; 3 Hospital Rothschild-AP-HP, 5, rue Santerre, 75012 Paris, France; bruno.frachet@gmail.com; 4 CEN, 18, rue P. Kergomard, 21000 Dijon, France; florent.herpin@groupecen.com (F.H.); florian.rigaudier@groupecen.com (F.R.)

**Keywords:** tinnitus, THI, tinnitus handicap inventory, permanent, fluctuating, food supplement, antioxidant

## Abstract

The aim of this study is to evaluate the efficacy of taking a daily supplement based on active compounds (AUDISTIM^®^ Day Night: A D/N) in alleviating tinnitus-related disability, as suggested by previous real-life studies. This double-blind randomized placebo-controlled study was conducted in adults with mild to severe tinnitus receiving a 3-month supplementation with A D/N (magnesium, vitamins, phytochemicals) or placebo (excipients without active ingredients). Tinnitus-related handicap (THI), psychological stress (MSP-9), and sleep quality (PSQI) were assessed at baseline and during intervention, perceived impression of tinnitus improvement at the end of the follow-up. The full set analysis included 114 patients (59 A D/N, 55 placebo) aged 53.8 ± 11.4 years, 58% women, with fluctuating (45%) or permanent (55%) tinnitus from 9.3 ± 9.4 years. A D/N supplementation led to greater changes in THI (−13.2 ± 16.0 vs. −6.2 ± 14.4, *p* = 0.0158, Cohen’s *d* = 0.44) at 3 months (primary outcome), especially with continuous tinnitus (−15.0 ± 16.3 vs. −4.6 ± 12.8, *p* = 0.0065), and, to a lesser extent, at 1 month (−9.8 ± 13.1 for A vs. −4.3 ± 12.1, *p* = 0.0213). PSQI significantly improved over time in both groups, but MSP-9 only with A D/N. In lines with previous observational studies, both clinical (THI score > 7 pts) and statistical (vs. placebo) improvement, more pronounced in permanent tinnitus, demonstrate the effectiveness of the combination of active compounds and support its use in the management of mild to severe tinnitus.

## 1. Introduction

Tinnitus has been defined in many ways, i.e., in the WHOS’s ICD11 [1] or in scientific literature [2,3,4], but not all of them capture its complex characteristics. To compensate for the lack of a complete, precise, and consensual definition of tinnitus, and to distinguish between tinnitus without suffering and tinnitus with associated suffering, a group of international experts recently proposed the following definitions: “*Tinnitus is the conscious awareness of a tonal or composite noise for which there is no identifiable corresponding external acoustic source, which becomes Tinnitus Disorder when associated with emotional distress, cognitive dysfunction, and/or autonomic arousal, leading to behavioral changes and functional disability*” [5]. Heterogeneity of forms, genetic and environmental etiological factors, associated condition or disease, and assessment for diagnosis make it challenging not only to specify accurate tinnitus prevalence over the general population but also to provide effective standardized treatments. However, with all types together, it has been estimated that around 740 million adults worldwide may be affected by tinnitus [6], and 65 million in European Union, meaning more than 1 in 7 adults in a gender-balanced way [7].

When extremely loud or bothersome, tinnitus is a major source of distress with well-established functional and psychological effects [8,9,10] and impaired quality of life [11,12]. Of adverse effects of tinnitus, sleep disturbance has been reported as the most common [13], but tinnitus can also affect daily activities or lead to depression and anxiety [3,14].

A wide range of drugs or non-pharmacological alternatives are offered to patients presenting with tinnitus [9,15,16,17]. Scientific reviews on tinnitus emphasize the absence of universally effective treatments, despite the high prevalence of this condition. Evaluated management options include daily sound therapy, cognitive-behavioral therapy, brain/neural stimulation, and medication (i.e., antidepressants, anticonvulsants, benzodiazepines/GABAergic drugs, glutaminergic drugs, muscle relaxants, sodium channel blockers, and others), but a deeper understanding of the underlying mechanisms is needed to develop optimal management strategies. Review of scientific data also highlights that there is no effective treatment for all patients with tinnitus [15,17,18]. As an example, a recent meta-analysis reported encouraging conclusions regarding the beneficial effects of drugs with brain-acting effects or anti-inflammatory/antioxidant effects on certain primary tinnitus [16]. In a recent double-blind, randomized, placebo-controlled study, an antioxidant-based dietary supplement was shown to be effective in reducing the discomfort and intensity of tinnitus [19].

Consistent results were reported in two observational studies which assessed the effectiveness of AUDISTIM Day/Night, a food supplement marketed since 2015 [20,21]. This supplement combines several active ingredients and antioxidants that may exert beneficial effects on tinnitus and related disorders. Claims of the different components of the food supplement are provided in detail in our previous works [20,21]. Conducted in France and Belgium, the two studies included a total of 421 patients with persistent subjective tinnitus enrolled by Ear Nose Throat practitioners in community settings or hospital facilities. Both studies displayed converging results in terms of the effectiveness of the supplement on reducing tinnitus-related impairment on daily life, psychological stress, and sleep quality. In both studies, tinnitus impairment on daily life was assessed using the THI (Tinnitus Handicap Inventory) [22], this questionnaire being the most frequently used in practice [18].

However, as pointed out by authors of the above-mentioned systematic reviews and meta-analyses, well-conducted randomized controlled trials that consider the subjective character of tinnitus are essential to assess efficacy of the interventions. As far as the effect of dietary supplements on tinnitus is concerned, we seem to be in the early stages of evidence-based medicine. The absence of a confirmatory randomized controlled trial, a heterogeneous intervention, or an evaluation of efficacy results in either no conclusions or inconsistent conclusions. Yet the absence of evidence does not mean there is not any. The present double-blind randomized controlled trial (RCT) aimed at evaluating the efficacy of A D/N on tinnitus in patients with mild to severe persistent tinnitus. The study methodology, as outlined by Kikidis et al. [23], follows the latest recommendations for RCTs in tinnitus. Changes in THI score at 3 months was chosen as primary endpoint, objective measurement such changes in hearing loss having been reported as not the most relevant outcome of treatment response [23]. The THI questionnaire, which is a validated in French version [24], is widely used tool for assessing efficacy in tinnitus, allowing for comparison between our work and others’.

## 2. Materials and Methods

Study design. This study was a double-blind, randomized, placebo-controlled with 1.1 ratio, parallel-arm, and monocentric. The study was set up at a clinical investigation center (CEN Experimental, Dijon, France) under principal investigator supervision. Having been duly informed about the study and in accordance with national provisions and the General Data Protection Regulation (GDPR), each patient gave written and informed consent to their participation in the study and to the processing of their personal data. After baseline data collection (M0), patients meeting all entry criteria were allocated to the A D/N and placebo groups. Patients took part in study assessments on three occasions, once at enrolment (visit M0) and twice during follow-up, i.e., after 1 (M1) and 3 months (M3) following the start of daily supplementation with their allocated intervention.

Participants. Patients aged 30 to 75 suffering from chronic subjective tinnitus, mild to severe, as assessed by a THI (Tinnitus Handicap Inventory) score of 12 to 76 [22], for at least 6 months could be included, as well as patients with ongoing pharmacological or alternative treatment (i.e., cognitive-behavioral, habituation, etc.) for their tinnitus could not be included. Patients presenting with catastrophic tinnitus (THI ≥ 78), hearing-head deafness, total implanted deafness, hearing disease (Menière’s disease, vestibular neuritis, neuroma, otosclerosis cholesteatoma, otitis including acute otitis media, otitis media with effusion and chronic suppurative otitis media, tympanic lesion, or cerumen plug), chronic metabolic or progressive disease, or psychiatric disorders (i.e., depressive episode, bipolar disorder). Patients were excluded if they had taken ototoxic treatments in the last 2 months (i.e., anti-inflammatories, anticoagulants, antiarrhythmics, hypotensive, antidepressants, monoamine oxidase inhibitors, benzodiazepines, or opioids). Shift workers and pregnant or breastfeeding women were also excluded.

Randomization and blinding. Eligible patients were assigned 1:1 to either A D/N or placebo. Randomization was generated using the block 4-randomization of the random function of the SAS software version 9.4 (SAS Enterprise Miner 13.1. SAS Institute Inc., Cary, NC, USA). All clinical investigation, data collection, assessments, and data analyses were blinded to randomization allocation.

Interventions. At M0 visit, depending on their allocated group, patients received 3 boxes of A D/N or placebo tablets containing 30 “Day” tablets and 30 “Night” tablets in blister packs, corresponding to 3 months’ supplementation. Supplementation should start the next day after M0 visit. AUDISTIM^®^ day/night consists of two “Day” and “Night” tablets to be taken daily in the morning and 30–60 min before bedtime, respectively. The Day tablet contained 515.6 mg of excipients including coating (maltodextrin, microcrystalline cellulose, sodium croscarmellose, magnesium stearate, silicon dioxide, hydroxypropylmethylcellulose, calcium sulfate, magnesium carbonate, hydroxypropylcellulose, stearic acid) and magnesium (124.5 mg), L-theanine (50.0 mg), *Ginkgo biloba* (40.0 mg), *Crataegus laevigata* (37.5 mg), quercetin (25.0 mg), and vitamins: nicotinamide (19.2 mg), cyanocobalamine (3.0 mg) (1.4 mg), pyridoxine (2.04 mg), and thiamine (1.68 mg). The Night tablet contained 483.1 mg of excipients including coating (same as Day plus Spirulina used as a colorant), *Melissa officinalis* L. (80.0 mg), magnesium (62.25 mg), *Ginkgo biloba* L. (40.0 mg), *Eschscholtzia californica Cham.* (40.0 mg), L-tryptophan (40.0 mg), zinc (31.95 mg), quercetin (25.0 mg), and melatonin (1.0 mg). Both tablets covered 100% of the French Recommended Daily Allowance [25] for vitamins and zinc, and 50% for magnesium. Placebo day and night tablets were indistinguishable from A D/N tablets and contained 742 mg of their respective excipients. Compliance with supplementation was assessed at M1 and M3.

Outcomes. THI score changes from baseline at 3 months was chosen as meaningful primary endpoint. The Tinnitus Handicap Inventory [22] and its French version [24] is a 25-item self-report measure to determine perceived tinnitus handicap severity which is classified as very mild (score 0–16), mild (score 18–36), moderate (score 38–56), severe (score 58–76), and catastrophic (78–90). A change in score of at least seven points has been considered to denote reliable clinically significant improvement on the THI [26]. Secondary efficacy criteria included changes from baseline at M1 and M3 of the subjective measures of psychological stress using MSP-9 (shorter nine-item version of the Psychological Stress Measurement) [27], with scores ranging from 9 to 72 (maximum stress), and sleep quality using the PSQI (Pittsburgh Sleep Quality Index) questionnaire [28] with scores ranging from 0 to 21 (poorer sleep quality). Patient were asked to subjectively assess their impression of tinnitus improvement using the PGII (Patient Global Impression of Improvement). Safety endpoints were assessed throughout the study.

Sample size. The sample size assumed a 10-point difference in the mean change at M3 in THI scores between A D/N and placebo. With a standard deviation of 15 [20,21], a reduction from baseline of 5 in the placebo arm and of 15 in the A D/N arm, at risk alpha = 0.05, with a power of 90 and in a bilateral situation, the sample size reached 98 subjects (49 per arm), increased to 110 subjects in total to account for dropouts.

Statistical analysis. Statistics were conducted according to the Statistical Principles for Clinical Trials ICH-E9 guidance [29], using SAS software version 9.4. Quantitative variables were described by mean and standard deviation (SD) or median and 95% confidence interval (95CI). Qualitative variables were described by number and percentage. Full set analysis was used as main efficacy analysis. Analyses were also conducted in the subsets of patients with fluctuant or permanent tinnitus. THI, MSP-9, and PSQI changes from baseline were compared between groups using a two-way (Time × Treatment) ANOVA, and their scores at M0, M1, or M3 using Chi^2^ test. Within the same group, M0, M1 and M3 scores were compared using paired *t*-test. Effect size was estimated using Cohen’s *d* [30], and related classification: small (*d*  = 0.2), medium (*d*  = 0.5), and large (*d* ≥ 0.8). PGII was compared between groups and subsets using Fisher test. The alpha risk for the significance level was set at 5%. There was no handling of missing data.

## 3. Results

### 3.1. Patient Disposition

**Figure 1:**
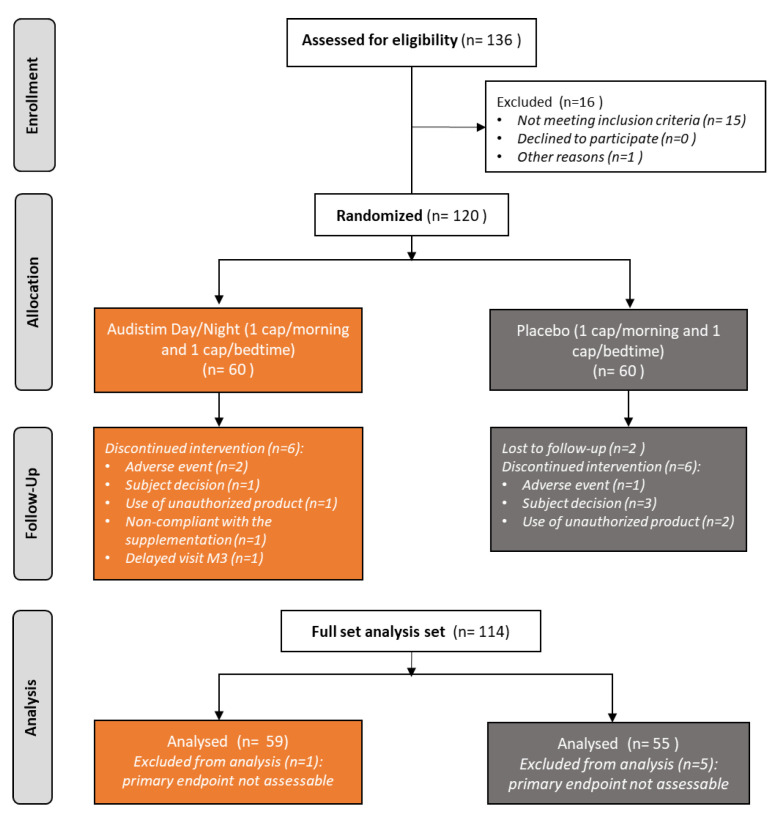
Study flow-chart.

From April 2022 to May 2023, 136 patients were screened at the clinical trial site, of whom 120 were randomized. The full analysis set (FAS) included 114 patients who all received interventions and had assessable primary endpoint (Figure 1). Patients were equally allocated to A D/N (n = 59) or placebo (n = 55) groups, thus respecting sample size assumptions. Attrition rates were lower than anticipated (20% vs. 3% in the placebo group and 10% in the A D/N group) and did not differ between groups (*p* = 0.2235).

### 3.2. Description of Patients and Tinnitus at Baseline

Baseline demographics and clinical characteristics of the patients did not differ between the A D/N and placebo groups (Table 1). Overall, patients (58.0% women) had a mean (SD) age of 53.8 (11.4) years, 94.7% (n = 112) did not present clinical abnormalities, and 68.4% (n = 78) no concomitant pathologies. As shown in Table 2, tinnitus characteristics did not differ between groups. Mean (SD) duration of tinnitus was 9.9 (10.5) years, and tinnitus was classified as permanent for 55.3% (n = 63) of patients and fluctuating for 44.7% (n = 51). In overall FAS, mean (SD) tinnitus-induced annoyance in daily life was rated 5.1 (1.7), corresponding to a moderate impact, and 67.5% (n = 77) reported associated symptoms (FAS results are not presented in Table 2). Mean (SD) THI score in FAS was 37.4 (16.9) of 100, and 34.2% (n = 39) and 13.2% (n = 15) had moderate to severe tinnitus-related handicap (FAS results are not presented in Table 2).

### 3.3. Changes in Tinnitus-Related Handicap under Dietary Supplementation

**Figure 2:**
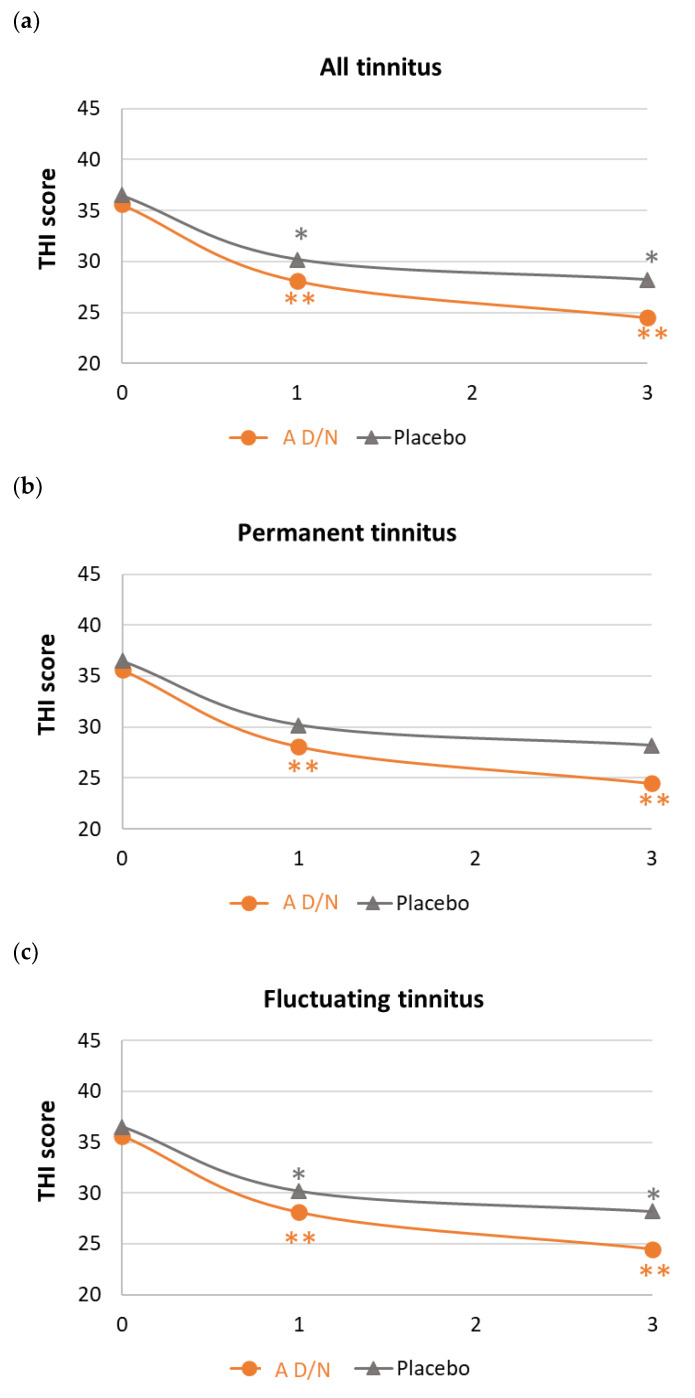
THI scores over 3 months of supplementation with A D/N (AUDISTIM Day/Night) or placebo: (**a**) all tinnitus (A D/N, n = 59; placebo, n = 55), (**b**) permanent tinnitus (A D/N, n = 32; placebo, n = 31), and (**c**) fluctuating tinnitus (A D/N, n = 27; placebo, n = 24). * and ** mean significant difference between M0 and M1 or M0 and M3 with paired *t*-test at *p* value <0.05 and <0.0001, respectively.

Changes from baseline at 3 months are presented in Table 3. Variation in THI score was significantly higher in the A D/N group vs. placebo (−13.2 ± 16.0 vs. −6.2 ± 14.4, *p* = 0.0158, Cohen’s *d* = 0.44). Greater improvement was observed in patients with permanent tinnitus (−15.0 ± 16.3 vs. −4.6 ± 12.8, *p* = 0.0065, Cohen’s *d* = 0.71). As suggested by Figure 2, to a lesser extent, significant variations of THI score were also reported from M1 in all patients (−9.8 ± 13.1 vs. −4.3 ± 12.1, *p* = 0.0213, Cohen’s *d* = 0.44) and patients with permanent tinnitus (−11.5 ± 13.6 vs. −2.7 ± 10.5, *p* = 0.0057, Cohen’s *d* = 0.72). A similar but not significant trend was observed in patients with fluctuating tinnitus (−7.8 ± 12.6 for vs. −6.3 ± 13.9, *p* = 0.6818, Cohen’s *d* = 0.11). Irrespective of the duration of intervention and type of permanent/fluctuating tinnitus, all variations in THI scores were above the minimum clinical significance of 7 points in the A D/N group, but never in the placebo group. With all cases of tinnitus combined, the rate of patients with at least a 20% reduction in THI score at M3 was significantly higher in the A D/N group. Probably due to the limited sample size of this subset, a higher but non-significant rate was reported in the subset of patients with permanent tinnitus.

### 3.4. Psychological Stress, Sleep Quality and Subjective Assessment of Tinnitus Improvement

#### 3.4.1. Psychological Stress and Sleep Quality

Evolution of psychological stress and sleep quality, assessed using MSP-9 and PSQI questionnaires, respectively, over the study, is detailed in Table 4. All patients had comparable moderate levels of stress, as suggested by MSP-9 score values [27]. MSP-9 scores were significantly lower by −3.9 (10.7) points at M3 (*p* = 0.008) compared to M0 in the A D/N group but not in the placebo. A similar trend was observed in patients under A D/N with permanent and fluctuating tinnitus. However, the modest variations from baseline at 3 months observed in the A D/N group did not achieve clinical significance for psychological stress reduction. At baseline, the mean FAS PSQI score was just above the threshold of 5 that separates good from bad sleepers, suggesting that the study population did not have significant sleep disorders. At the end of the follow-up, PSQI scores were modestly but significantly lower in all patients, except in placebo patients with permanent tinnitus.

#### 3.4.2. Subjective Assessment

**Figure 3:**
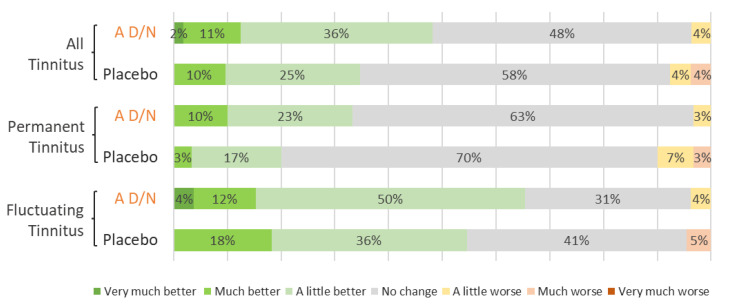
Patient subjective assessment of improvement of tinnitus after 3 months of supplementation with A D/N (AUDISTIM Day/Night) or placebo, all tinnitus (A D/N, n = 59; placebo, n = 55), permanent tinnitus (A D/N, n = 30; placebo, n = 30), and fluctuating tinnitus (A D/N, n = 27; placebo, n = 24).

Subjective assessments of tinnitus improvement reported by the patients are presented in Figure 3. A non-significant trend in favor of the A D/N was observed in both subsets of patients with permanent tinnitus and with fluctuating tinnitus. However, while 65% (n = 17) in the A D/N group vs. 55% (n = 12) in the placebo group of patients with fluctuating tinnitus reported improvements, 33% (n = 10) in the A D/N group vs. 20% (n = 6) in the placebo group of patients with permanent tinnitus also reported improvements.

### 3.5. Safety

During intervention, 15% (n = 9) patients in the A D/N group and 18% (n = 11) in the placebo group experienced adverse events with relationship to treatment classified as possible or probable by the investigator. In the placebo group, 6 of the 12 adverse events were tinnitus worsening. One of the adverse events (mild intermittent nausea with probable relation to the placebo) led to study discontinuation. In the A D/N group, 4 of 11 adverse reactions were gastrointestinal troubles. Others including headache (n = 2) or insomnia (n = 2) were also reported by placebo patients. Of them, two adverse events (mild acute headache with probable relation to the supplement and mild persistent epigastralgia with possible relation to the supplement) led to study discontinuation.

## 4. Discussion

Over the past decade, tinnitus has become one of the most common causes of disability [31]. As mentioned in the introduction, despite the discomfort and distress, some patients remain without effective treatment. It is becoming increasingly clear that a lack of a treatment is not an acceptable solution for patients, and that all attempts to improve tinnitus and relieve related symptoms and suffering will benefit patients. AUDISTIM^®^ Day/Night was developed with this in mind. Thanks to its combination of active compounds, this food supplement aims to offer a non-pharmacological treatment for subjective tinnitus of various etiologies.

One of the main findings of this RCT is that results confirmed the effectiveness of AUDISTIM^®^ Day/Night on tinnitus-related impairment in daily life reported in the two real-world studies in patients suffering from chronic subjective tinnitus [20,21]. Results also support the favorable effect of the food supplement on psychological stress and sleep quality, as already observed in the two observational studies, although statistical significance was not always achieved for both these criteria. Another finding of interest of the current RCT is that response to the supplementation was greater in patients with permanent compared to fluctuating tinnitus.

In the French and Belgian observational studies, THI scores were significantly decreased by −17.9 points and −9.9 points, respectively, vs. −13.2 points in patients of the present RCT consuming A D/N. Thus, all changes in THI scores under A D/N supplementation almost doubled in clinical significance by 7 points according to Zeeman’s criteria [26] whereas those under the placebo did not. These differences in THI changes between studies may be explained by patient tinnitus heterogeneity. As an example, only the Belgian study included patients with extremely severe tinnitus based on THI scores indicating “catastrophic handicap”, in which poor response to treatment is expected. In the French study, the mean (SD) baseline THI score was close to that (44.6 (23.4)) of the present RCT (38.9 (16.2)), but changes in THI score were slightly higher. It cannot be ruled out that selection and/or evaluation bias, inherent in observational studies, may have led to an overestimation of the real effectiveness. As the two observational studies did not specify Newman’s criteria (percent of patients with 20% increase in THI [22]), no comparison with the present study is possible.

AUDISTIM^®^ Day/Night is a combination of nutrients, phytochemicals, and plant extracts with recognized antioxidant properties or nutritional claims. Beneficial effects of antioxidant association were also shown by other in patients of similar profiles to those of this RCT. In Petridou’s RCT, taking multivitamin and mineral tablets once a day and alpha-lipoic acid tablets twice a day for three months induced a non-clinical but significant 6.1-point reduction in THI scores [19]. However, there was no significant difference with the placebo, probably due to a strong response in this arm. Another randomized controlled trial reported a 10.0-point reduction in THI score after 3 months’ consumption of 100 mg of the higher antioxidant-rich extract of *Euterpe oleracea martius* (Açaí) once a day [32]. Overall, these results provide clinical evidence for the use of antioxidant molecules in tinnitus that should not be ignored. The dietary supplement studied contains *Ginko biloba*, the effectiveness of which on tinnitus is still uncertain, as concluded by the latest Cochrane review [33]. It should be noted that the poor quality (i.e., small samples, unexplained high drop-out rate, methodological limitations, including an unidentified primary outcome and high risk of bias, which led authors’ review to classify the certainty of the evidence as low to very low) of the 12 RCTs included in this systematic review meant that no further conclusions could be drawn. The same could be said of zinc’s controversial efficacy. The supplement also contains melatonin, for which promising beneficial effects on tinnitus have been reported [34], as well as antioxidant activity [35]. It is well established that melatonin helps people fall asleep and relieves sleep disorders as well as lemon balm (*Melissa officinalis*), thanks to its traditional use to relieve mild symptoms of mental stress and to promote sleep [36]. Based on PSQI scores, the patients in our study had only mild sleep disturbances. The modest variations in sleep quality alone cannot account for the variations in THI scores. We can therefore assume that sleep-inducing ingredients such as melatonin and extract of lemon balm leaves are not the active ingredients of A D/N that have played a major role in improving tinnitus severity. Studies carried out on patients with markedly poor sleep quality could help support this hypothesis. To date, the three studies carried out on the food supplement do not make it possible to determine whether the effect observed is due to certain compounds alone or whether the effects have been potentiated. However, their combination appears to be more effective than monotherapy with *Ginko biloba*, each of antioxidants (i.e., plant extract or quercetin), melatonin, vitamin B12, or zinc [19,33,34,35,36]. We also assume that antioxidants play a major role as they may exert a synergic activity.

While experts have stressed the need to separately evaluate responses to intervention in patients with chronic/persistent tinnitus, meaning lasting at least 3 months [5], either continuously perceived or with intermittent occurrence, very few studies have focused on it [23]. This RCT differ from others as the efficacy of AUDISTIM^®^ Day/Night supplementation was investigated in two subsets of the FAS, namely patients with permanent or fluctuating tinnitus. In the absence of a well-defined wording and to avoid confusion with transient ear noise, in the present study, tinnitus lasting all day or present almost all the time was classified as “permanent”, while tinnitus over 5 min in duration which occurs occasionally was classified as “fluctuating”. The results of our study showed that the variation in THI scores from baseline at 3 months was greater in patients with permanent tinnitus, suggesting that supplementation is beneficial for patients with greater day-to-day tinnitus-related discomfort or suffering. Results also highlighted that the placebo effect was lower in patients with permanent tinnitus than in those with fluctuating tinnitus. Higher placebo-effect and lower effectiveness of the supplementation may explain why variations in THI scores did not differ between A D/N and placebo in this subset.

Similar figures were obtained when sleep quality was assessed in both subsets. In patients with permanent tinnitus, sleep quality improved significantly, but not in the placebo group. Interestingly, the PSQI was below the threshold that distinguishes “good sleepers” from “bad sleepers” at the end of the A D/N intervention [28]. By contrast, in patients with fluctuating tinnitus, sleep quality was significantly and comparably improved in patients taking the supplement or placebo. As well as sleep quality, which was more impaired in patients with fluctuating tinnitus, psychosocial stress was also greater in these patients. In line with the results of the two observational studies, regardless the type of tinnitus, psychosocial stress was improved in patients receiving the supplementation but not the placebo. Significance was only reported on overall patients of the A D/N group, suggesting that the small size of the subsets failed to achieve statistical significance.

Patients’ subjective assessment of tinnitus improvement also showed a trend in favor of the A D/N over placebo. Among patients with permanent tinnitus, only a third perceived a positive change in tinnitus, while THI score increased by at least 20% in three-quarters of patients, and sleep quality for most of them. In contrast, the results from one assessment to the next were consistent in patients with fluctuating tinnitus. This observation is hardly surprising, and once again raises the difficulties of evaluating the efficacy of tinnitus intervention in this condition of highly subjective nature [9,17,23]. In this context, it is worth remembering that the Tinnitus Handicap Inventory, used in this RCT, remains a validated tool with predefined clinical meaning, and that clinically significant effect was achieved with A D/N supplementation in overall patients as well as patients with permanent or fluctuating tinnitus [26,37].

Finally, data from the present study also show the safe profile of the supplement. No serious adverse reactions were reported, in line with food ingredient intervention data mentioned in systematic reviews [18,33]. While gastrointestinal troubles or headaches have been described in *Ginko biloba* studies, the pooled results showed no significant difference in the occurrence of these events compared to the placebo [33]. In this study, few adverse events, including gastrointestinal troubles or headaches, were reported, with doubtful relationship to the food supplement as it appeared not possible to clearly state that they were side effects.

This study has some limitations (i.e., monocentric study, follow-up limited to 3 months, and eligibility criteria which may mean that conclusions cannot be extrapolated to patients without the same characteristics). The first is that the sample size proved too small to achieve statistical significance when variations from baseline in the secondary endpoints were compared between the A D/N and placebo groups. Sample size calculation assumptions were based on THI changes, chosen as the primary endpoint, measured in the two observational studies. Even though the sample size usually does not consider the secondary endpoints, such a high placebo effect was not anticipated. This high placebo effect may also explain the moderate sample effect on primary endpoint. The second limitation is that confounded factors such as mood disorders or anxiety, as well as temporomandibular disorders [38], were not collected. The groups being comparable at enrollment, it is assumed that randomization and blinding have limited this risk of bias. Finally, annoyance using VAS as part of perceptual aspects of tinnitus was only collected at baseline as part of the evaluation of tinnitus severity, but any changes were not investigated during the intervention. Despite these limitations, we are reasonably confident with the conclusions of the study.

## 5. Conclusions

In line with the two observational studies, significant clinical and statistical improvement over placebo suggest the effectiveness of AUDISTIM^®^ Day/Night on tinnitus severity. The combination of active compounds is supposed to be more effective in permanent tinnitus. Beneficial trends on psychological stress and sleep quality deserve to be investigated in further studies, as well as long-term effects on tinnitus-related handicap. To conclude, the results of the present study support the use of AUDISTIM^®^ Day/Night in the management of mild to severe tinnitus.

## Figures and Tables

**Table 1:** Description of patients (n = 114).

	A D/N (n = 59)	Placebo (n = 55)	*p*-Value
**Age** (years)			
Mean (SD)	54.6 (12.5)	52.9 (10.1)	0.4281 *
Median (IQR)	59.0 (43.0 65.0)	53.0 (44.0; 59.0)	
**Gender** (% male/% female)	37.3/62.7	47.3/52.7	0.2806 **
**Concomitant disorder** (Yes) (n (%))	23 (39.0)	13 (23.6)	0.1693 **
**Clinical abnormalities** (Yes) (n (%))	1 (1.7)	5 (9.1)	0.1047 **

* Student paired test, ** Chi-squared test or Fisher test.

**Table 2:** Description of tinnitus at baseline (n = 114).

	A D/N (n = 59)	Placebo (n = 55)	*p*-Value
**Duration** (years)			
Mean (SD)	9.5 (9.7)	9.0 (9.3)	0.7862 *
Median (IQR)	6.0 (3.0; 13.0)	5.0 (2.0; 15.0)	
**Main etiology**			
Idiopathic (n (%))	25/42.4	18/32.7	0.7529 **
Acoustic trauma (n (%))	18/30.5	20/36.4	0.5075 **
ENT/Viral infection (n (%))	6/10.2	6/10.9	0.7529 **
Presbycusis (n (%))	5/8.5	2/3.6	0.4404 **
Vascular disorders (n (%))	2/3.4	5/9.1	0.2597 **
**Location**			
Both ears (n (%))	37/62.7	38/69.1	
Right or Left ear (n (%))	19/32.2	17/30.9	0.2105 **
Head (n (%))	3/5.1	0/0.0	
**Tinnitus onset**			
Permanent (n (%))	32/54.2	31/56.4	0.8195 **
Fluctuating (n (%))	27/45.8	24/43.6	
**Associated symptoms (Yes) (n (%))**	41/69.5	36/65.5	
Hypoacusis/hyperacusis	25/42.4	25/45.5	0.8885 **
Headache	13/22.0	8/14.5	0.3027 **
**Annoyance (0–10 VAS)**			
Mean (SD)	5.3 (1.5)	4.9 (1.8)	0.1913 *
Median (IQR)	5.0 (4.0; 6.7)	5.0 (3.0; 7.0)	
**THI total score**			
Mean (SD)	38.9 (16.2)	35.7 (17.5)	0.3180 *
Median (IQR)	36.0 (26.0; 54.0)	30.0 (22.0; 48.0)	
**Tinnitus classification**			
No handicap (n (%))	7 (11.9)	7 (12.7)	0.6329 **
Mild handicap (n (%))	23 (39.0)	23 (41.8)	
Moderate handicap (n (%))	23 (39.0)	16 (29.1)	
Severe handicap (n (%))	6 (10.2)	9 (16.4)	
Catastrophic handicap (n (%))	0 (0.0)	0 (0.0)	

THI: Tinnitus Handicap Inventory questionnaire, ENT: ear, nose throat infection or viral infection by SARS-Cov2. * Student paired test, ** Chi-squared test or Fisher test.

**Table 3:** Changes in tinnitus-related handicap at 3 months.

	A D/N	Placebo	*p*-Value	Cohen’s *d*
All tinnitus (n = 104)	(n = 59)	(n = 55)		
Changes in THI scores				
Mean (SD)	−13.2 (16.0)	−6.2 (14.4)	0.0158 *	0.44
95%CI	−17.4/−9.1	−10.1/−2.3		
THI score reduction ≥20%				
n (%)	40 (67.8)	26 (47.3)	0.0266 **	
Permanent tinnitus (n = 63)	(n = 32)	(n = 31)		
Changes in THI scores				
Mean (SD)	−15.0 (16.3)	−4.6 (12.8)	0.0065 *	0.71
95%CI	−20.9/−9.1	−9.3/0.1		
THI score reduction ≥20%				
n (%)	23 (71.9)	15 (48.4)	0.0568 **	
Fluctuating tinnitus (n = 51)	(n = 27)	(n = 24)		
Changes in THI scores				
Mean (SD)	−11.1 (15.7)	−8.3 (16.3)	0.5384 *	0.17
95%CI	−17.3/−4.9	−15.2/−1.5		
THI score reduction ≥20%				
n (%)	17 (63.0)	11 (45.8)	0.2198 **	

* ANOVA time x treatment, ** Chi-squared test or Fisher test.

**Table 4:** Psychological stress and sleep quality.

	A D/N			Placebo			A D/N vs. Placebo	
	Baseline Mean (SD)	Final Mean (SD)	*p*-Value *	Baseline Mean (SD)	Final Mean (SD)	*p*-Value *	*p*-Value **	Cohen’s *d*
All tinnitus	(n = 56)			(n = 52)				
MSP-9 scores	35.4 (12.6)	31.4 (11.2)	0.008	34.1 (11.2)	32.2 (11.1)	0.1947	0.3190	0.19
PSQI scores	7.4 (3.5)	6.0 (3.2)	0.0014	7.3 (3.5)	6.3 (3.0)	0.0374	0.3979	0.16
Permanent tinnitus	(n = 30)			(n = 30)				
MSP-9 scores	33.5 (10.4)	29.4 (9.8)	0.0655	32.5 (10.8)	31.8 (11.1)	0.6561	0.2062	0.33
PSQI scores	6.7 (3.6)	5.3 (2.5)	0.0154	6.4 (3.4)	6.0 (3.4)	0.4077	0.1855	0.33
Fluctuating tinnitus	(n = 26)			(n = 22)				
MSP-9 scores	37.5 (14.5)	33.7 (12.4)	0.0586	36.1 (11.5)	32.6 (11.4)	0.2026	0.9359	0.03
PSQI scores	8.2 (3.3)	6.7 (3.8)	0.0385	8.4 (3.3)	6.8 (2.3)	0.0458	0.9128	0.03

* Student paired test, ** ANOVA time X treatment.

## Data Availability

The data that support the findings of this study are available from CEN (contact@groupecen.com), upon reasonable request.
